# The complete chloroplast genomes of *Rhamnus arguta* Maxim. and *R. parvifolia* Bunge (Rhamnaceae)

**DOI:** 10.1080/23802359.2026.2728195

**Published:** 2026-09-09

**Authors:** Dongting Lu, Xin Jin, Xingwang Zhang, Chan Zhang, Xianfeng Jiang, Yanping Xie

**Affiliations:** ^a^ School of Life Sciences, Huaibei Normal University, Huaibei, China; ^b^ School of Life Sciences, Henan Normal University, Xinxiang, China; ^c^ College of Agriculture and Bioscience, Dali University, Dali, China

**Keywords:** Chloroplast genome, *Rhamnus*, Rhamnaceae, phylogenetic analysis, genome characterization

## Abstract

The genus *Rhamnus* L. (Rhamnaceae) has high medicinal, ecological, and ornamental value, but its infrageneric classification remains unclear. Here, we sequenced, assembled, and annotated the complete chloroplast genomes of *Rhamnus arguta* and *R. parvifolia* using Illumina sequencing. Both plastomes exhibit the typical quadripartite structure, with lengths of 160,566 bp and 161,243 bp, containing 128 and 129 genes, respectively. Phylogenetic analyses support the monophyly of *Rhamnus* and its close relationship with *Frangula*. This study provides genomic resources for further phylogenetic and comparative studies within Rhamnaceae.

## Introduction

The genus *Rhamnus* L. (Rhamnaceae) is widely distributed across temperate to tropical regions of eastern and southwestern Asia, southwestern and northern America (Mabberley 1987). This genus comprises approximately 150 species and has a long controversial taxonomic history and can thus be regarded as an example of a cosmopolitan genus in Rhamnaceae (Richardson et al. 2000). Species of *Rhamnus* are valued for their medicinal properties, including antioxidant, anti-inflammatory, and anti-mutagenic effects. Additionally, they are used as tea substitutes, sources of fatty oils and ornamental plants. Their well-developed root systems and adaptability to various environments make them crucial for soil and water conservation, windbreaks, and sand fixation, significantly contributing to ecological stability (Gassmann et al. 2008). Given their ecological and economic importance, comprehensive genomic resources are essential for clarifying evolutionary relationships and conservation-related studies.

*Rhamnus arguta* Maxim. 1866 and *R. parvifolia* Bunge 1833 are two small deciduous shrubs mainly distributed in East Asia. *R. arguta* usually grows as a relatively tall shrub or small tree with sparse or absent branch spines, large ovate-cordate leaves and long petioles. In contrast, *R. parvifolia* is a dwarf shrub characterized by well-developed branch spines, small rhombic-elliptic leaves and short petioles. Such morphological characteristics of *R. parvifolia* may reflect adaptation to relatively dry and disturbed habitats. Although plastome sequences are available for several Rhamnaceae genera (Hauenschild et al. 2016; Shi et al. 2024), genomic resources for *Rhamnus* remain limited, with complete chloroplast genomes reported for only a few species (Xie et al. 2020). Compared with previously published *Rhamnus* plastomes, *R. arguta* and *R. parvifolia* represent distinct morphological and ecological forms within the genus, and further expand the taxonomic sampling of available plastome data. Here, we report the complete chloroplast genome sequences of these two species, enriching the plastome resources of *Rhamnus* and providing valuable data for further phylogenetic and evolutionary studies within this taxonomically complex genus.

## Materials and methods

### Plant material, DNA extraction, and sequencing

**Figure 1.**
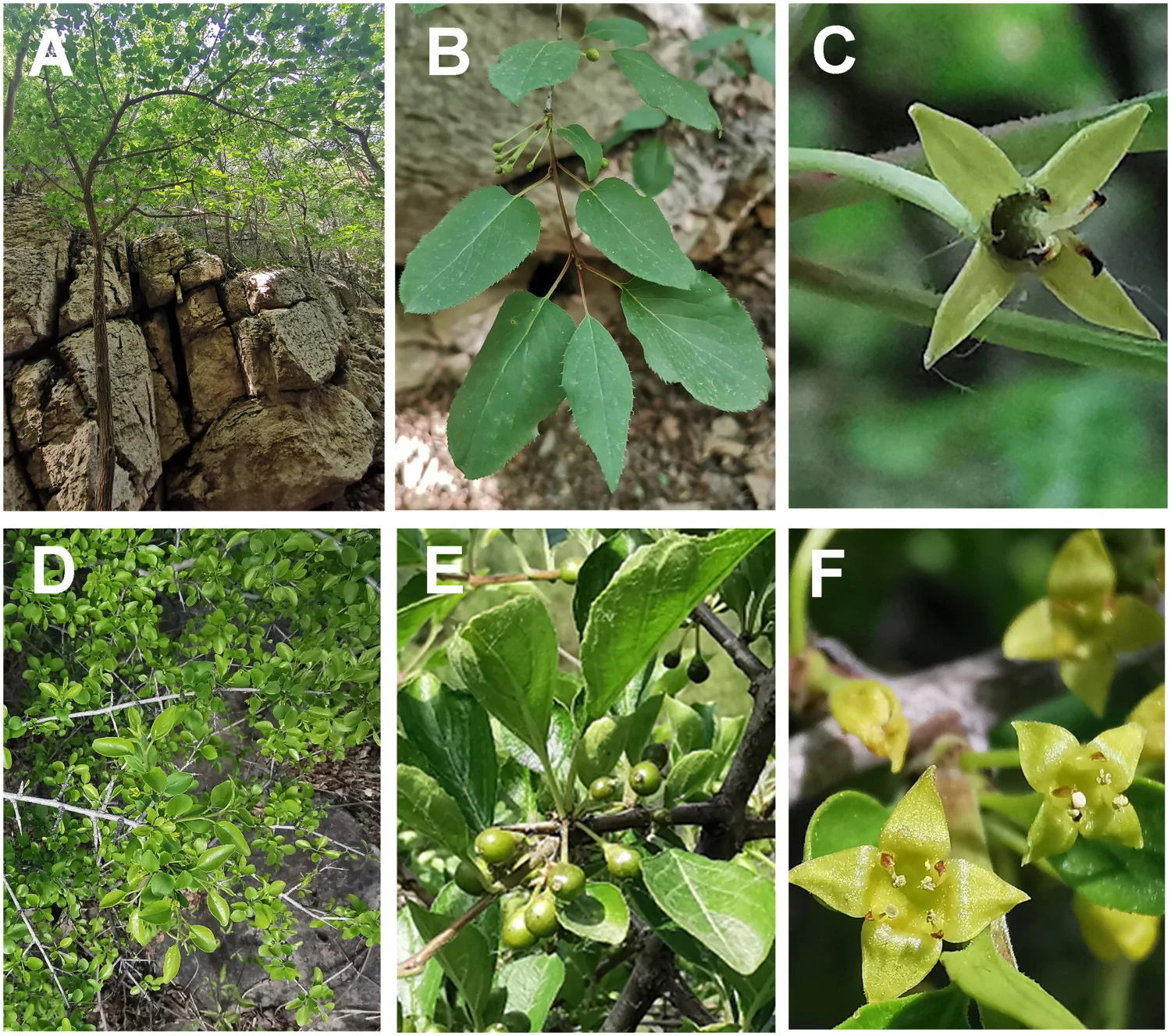
Field morphology of (A–C) *Rhamnus arguta* and (D–F) *R. parvifolia. R. arguta* has subopposite, ovate-cordate leaves with densely serrulate, sharp-toothed margins, 1–3 cm-long petioles, and yellowish-green tetramerous flowers; pistillate flowers possess a three-lobed stigma. By contrast, *R. parvifolia* bears rhombic-elliptic leaves clustered on short branchlets, 4–15 mm-long petioles, finely crenate-serrulate leaf margins, and yellowish-green tetramerous flowers. The photographs were taken by Yanping Xie.

The fresh leaves of *R. arguta* and *R. parvifolia* (Figure 1) were collected from Huangcangyu (32.34°N, 117.99°E) and Huaibei (34.06°N, 117.07°E), Anhui Province, China. The specimens were deposited in the Key Laboratory of Plant Resource and Biology in Huaibei Normal University (Yanping Xie, xieyanping0001@163.com), under voucher accession numbers XYP2024136 and XYP2024139. The specimens were identified as *R. arguta* and *R. parvifolia* based on diagnostic morphological characters, including leaf arrangement, leaf shape, margin morphology, and petiole length. Total genomic DNA was extracted from silica-gel-dried plant leaves using the HiPure SF Plant DNA Mini Kit (Guangzhou Magen Biotechnology Co., Ltd., Guangzhou, China). DNA was assessed for quality and randomly fragmented into 200–500 bp inserts. Sequencing libraries were constructed using 300 ng of genomic DNA and sequenced on the Illumina NovaSeq X platform (Xuzhou Yangrui Biotechnologies Co., Ltd., Xuzhou, China), generating 150 bp paired-end (PE150) reads.

### Genome assembly and annotation

After filtering adapter sequences and low-quality reads using Cutadapt v1.16 (parameters: -q 20 -m 20), the chloroplast genome was assembled using NOVOPlasty v4.3.1 (Dierckxsens et al. 2017). Genome annotation was performed using PGA v. C2019 (Qu et al. 2019), with the chloroplast genome of *R. globosa* (GenBank accession number ON009012) used as the reference. The annotated protein-coding genes were manually checked and corrected where necessary. The circular genome maps were drawn using the online tool CPGview with default parameters (http://47.96.249.172:16085/cpgview/drawmap). Finally, the annotated results were deposited in GenBank under accession numbers PQ433219 (*R. arguta*) and PQ433220 (*R. parvifolia*).

### Phylogenetic analysis

To investigate the phylogenetic relationship of Rhamnaceae, 28 additional complete chloroplast genome sequences were obtained from NCBI and aligned using MAFFT v7.307 (Katoh and Standley 2013) under default parameters. Two species from Elaeagnaceae (*Hippophae rhamnoides* and *Elaeagnus macrophylla*), a sister lineage to Rhamnaceae, were selected as outgroups (Choi et al. 2015; Hauenschild et al. 2016). A maximum-likelihood phylogenetic tree was reconstructed by online RAxML BlackBox software (Stamatakis et al. 2008) under the GTR+G substitution model, and branch support was assessed using 1000 bootstrap replicates.

## Results

**Figure 2.**
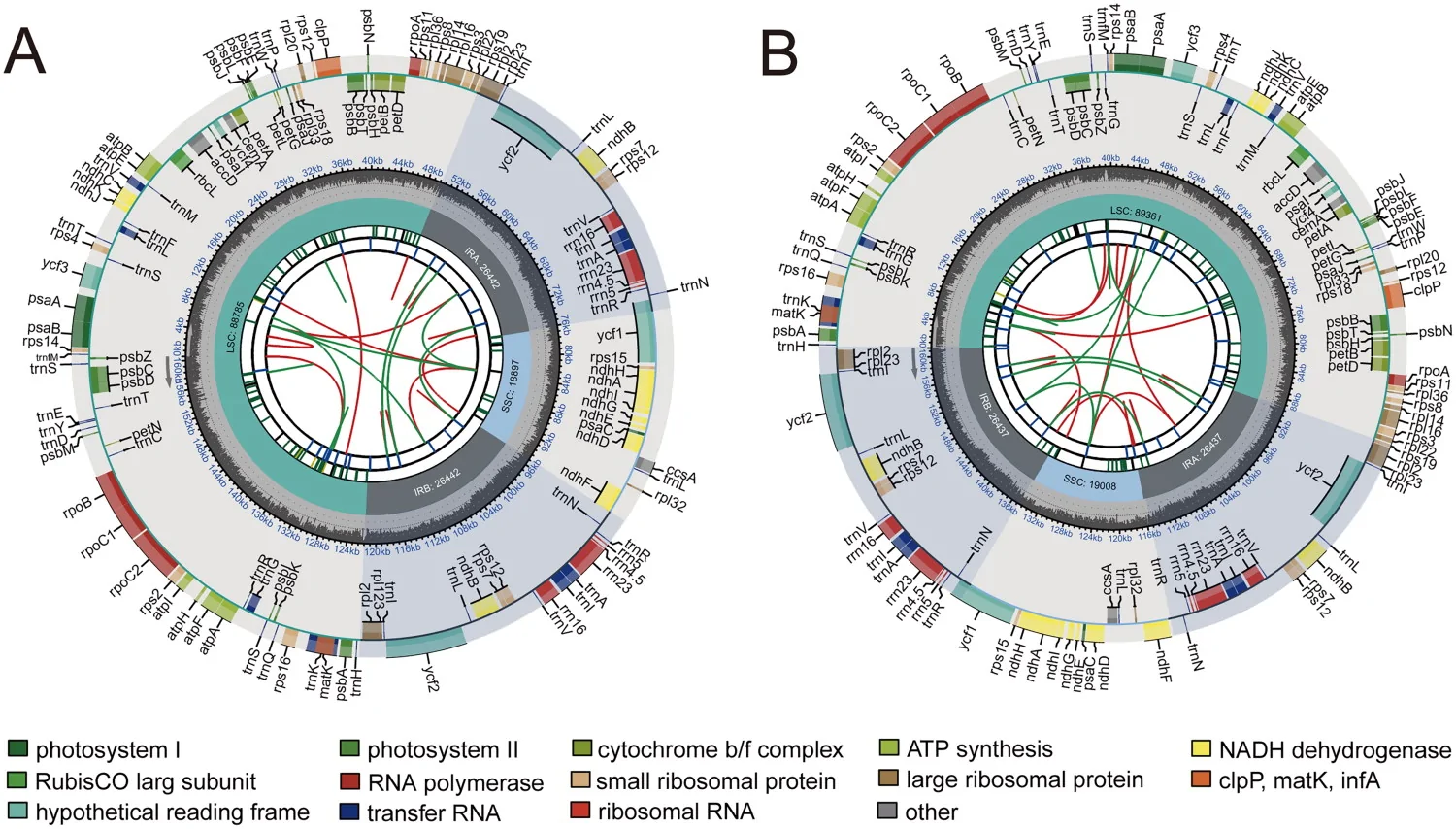
The chloroplast genome maps of (A) *Rhamnus arguta* and (B) *R. parvifolia.* The maps highlight genes transcribed in a clockwise direction within the circle and counterclockwise outside the circle. Genes from the same functional category are color-coded, as detailed in the figure legends. The darker gray columns in the inner circle represent GC content.

The average sequencing depth for *R. arguta* and *R. parvifolia* was greater than 3000×, with coverage close to 100% (Supplementary Figure S1). After assembling the chloroplast genomes of *R. arguta* and *R. parvifolia*, both genomes exhibited a typical quadripartite structure (Figure 2). The total length of the chloroplast genome for *R. arguta* was 160,566 bp, while that of *R. parvifolia* was 161,243 bp. The structure and composition of their chloroplast genomes are summarized in Table 1. The base compositions of the chloroplast genome were uneven in different regions for both species (Table 1). The chloroplast genomes of *R. arguta* and *R. parvifolia* exhibited largely similar gene organization, with *R. parvifolia* containing one additional gene. In both species, only *clp*P, *rps*12, and *ycf*3 contained two introns, whereas all other intron-containing genes contained a single intron. The analysis of the genome annotation results further confirmed the accuracy of the annotations for both cis- and trans-splicing genes (Supplementary Figure S2).

**Table 1.** Summary of chloroplast genome of *Rhamnus arguta* and *R. parvifolia.*

Genome features	Length (bp) | GC content (%)				Genes (unique gene number)			
	Cp genome	LSC	SSC	IR	Genes	Protein coding genes	tRNAs	rRNAs
*R. arguta*	160,566 | 37.16	88,785 | 35.07	18,897 | 31.39	26,442 | 42.75	128 (111)	84 (78)	36 (29)	8 (4)
*R. parvifolia*	161,243 | 37.12	89,361 | 35.00	19,008 | 31.39	26,437 | 42.76	129 (112)	84 (78)	37 (30)	8 (4)

Unique genes exclude duplicated copies in the IR regions.

**Figure 3.**
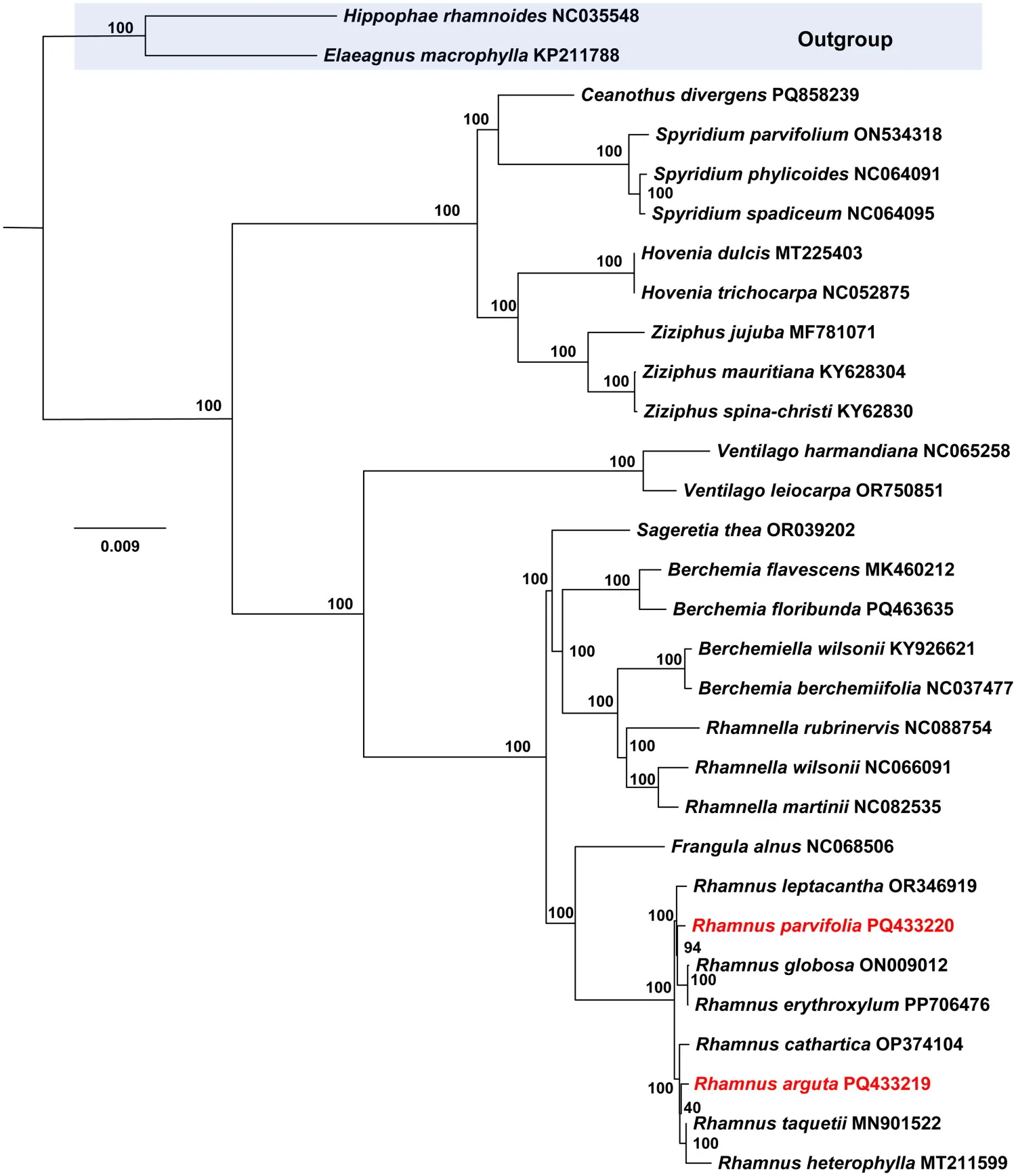
The maximum-likelihood (ML) phylogenetic tree reconstructed based on chloroplast genome sequences from Rhamnaceae and two outgroup species from Elaeagnaceae. The bootstrap support value is labeled for each node. The *Rhamnus parvifolia* (PQ433220) and *R. arguta* (PQ433219) highlighted in red were sequenced in this study. The additional sequences used for tree construction are as follows: *R. heterophylla* MT211599, *R. taquetii* MN901522, *R. cathartica* OP374104, *R. erythroxylum* PP706476 (unpublished), *R. globosa* ON009012, *R. leptacantha* OR346919, *Frangula alnus* NC068506, *Rhamnella martinii* NC082535, *Rh. wilsonii* NC066091, *Rh. rubrinervis* NC088754 , *Berchemia berchemiifolia* NC037477, *B. wilsonii* KY926621, *B. floribunda* PQ463635, *B. flavescens* MK460212, *Sageretia thea* OR039202, *Ventilago leiocarpa* OR750851, *V. harmandiana* NC065258, *Ziziphus spina-christi* KY628305, *Z. mauritiana* KY628304, *Z. jujuba* MF781071, *Hovenia trichocarpa* NC052875, *H. dulcis* MT225403, *Spyridium spadiceum* NC064095, *S. phylloides* NC064091, *S. parvifolium* ON534318, *Ceanothus divergens* PQ858239, and outgroup: *Elaeagnus macrophylla* KP211788 and *Hippophae rhamnoides* NC035548. The accession numbers are listed following the respective species names, the corresponding references are provided in Supplementary Table S1 at the end of the supplementary document.

The phylogenetic tree of Rhamnaceae (Figure 3) revealed that the genus *Rhamnus* is a monophyletic group based on the included samples. Both *R. arguta* and *R. parvifolia* were placed within the *Rhamnus* clade, although support for some internal relationships was relatively low. The genus *Frangula* is the closest genus to *Rhamnus* with very high bootstrap values.

## Discussion and conclusions

In this study, we report the complete chloroplast genomes of *R. arguta* and *R. parvifolia* for the first time. Both chloroplast genomes exhibit a typical quadripartite structure that is conserved across many plant species. The plastome of *R. parvifolia* (161,243 bp) is 677 bp longer than that of *R. arguta* (160,566 bp), reflecting modest genome size variation commonly reported within Rhamnaceae family and other angiosperms (Shi et al. 2024; Cauz-Santos et al. 2025). The observed genome size difference is associated with variation in the lengths of the LSC, SSC, and IR regions, which may reflect minor boundary shifts commonly observed among angiosperm plastomes (Sun et al. 2024; Cauz-Santos 2025; Zheng et al. 2025). The high structural conservation observed between the two species suggests a relatively conserved plastome organization despite their ecological breadth.

Previous phylogenetic studies of Rhamnaceae have primarily relied on morphological characters and a limited number of nuclear and chloroplast DNA markers, which have inherent limitations (Richardson et al. 2000; Hauenschild et al. 2016). In contrast, complete chloroplast genome sequences have proven highly effective for phylogenetic reconstruction in many plant groups (Lao et al. 2024; Cauz-Santos et al. 2025). In the present study, phylogenetic analysis based on complete chloroplast genome sequences yielded a well-supported topology for Rhamnaceae. The genus *Rhamnus,* which comprises approximately 150 species, was recovered as monophyletic, with *Frangula* as its closest relative, consistent with previous studies (Hauenschild et al. 2016, 2018). Both *R. arguta* and *R. parvifolia* were placed within the *Rhamnus* clade. However, relationships among closely related *Rhamnus* species remain incompletely resolved. The short branch lengths among *Rhamnus* species suggest limited plastome sequence divergence within the genus. The low support for some internal nodes may reflect limited phylogenetically informative variation and taxon sampling. These results provide additional plastome resources for *Rhamnus* and contribute to future studies of phylogenetic relationships within Rhamnaceae.

Beyond phylogenetic reconstruction, the generation of complete plastome resources contributes to broader genomic datasets that may support future research. Although *Rhamnus* is not a major crop genus, recent studies have emphasized the importance of integrating genomic resources into crop improvement and sustainable utilization strategies (Zafar et al. 2024; Manzoor et al. 2025). This study presents the first complete chloroplast genome sequences of *R. arguta* and *R. parvifolia*, which are structurally conserved and differ slightly in genome size, which is associated with variation in the lengths of the LSC, SSC, and IR regions. Phylogenomic analysis provides improved resolution within Rhamnaceae. These newly generated plastome resources enrich genomic data for *Rhamnus* and provide a foundation for future studies.

## Supplementary Material

Supplementary_Materials_Revised0618.docx

## Data Availability

The original sequencing data have been submitted to the NCBI database (https://www.ncbi.nlm.nih.gov) and have been assigned GenBank accession numbers: PQ433219 (*R. arguta*) and PQ433220 (*R. parvifolia*). The associated BioProject, SRA, and Bio-Sample numbers are PRJNA1231193, SRR32571926–SRR32572988, and SAMN47203518–SAMN47217406.
