# Supplementary material for: The complete chloroplast genomes of Rhamnus arguta Maxim. and R. parvifolia Bunge (Rhamnaceae)
Source: Mitochondrial DNA B Resour. 2026 Sep 9;11(10):1134–9. doi: 10.1080/23802359.2026.2728195 (PMC13560404; doi:10.1080/23802359.2026.2728195)
Supplement: Supplementary file 1 [file TMDN_A_2728195_SM3855.docx]

The complete chloroplast genomes of *Rhamnus arguta* Maxim. and *R. parvifolia* Bunge (Rhamnaceae)

Figure S1. Coverage depth figure of the *Rhamnus arguta* and *R. parvifolia* chloroplast genomes.

Figure S2. The schematic diagrams of cis- and the trans-splicing genes in two *Rhamnus* chloroplast genomes. (a) Cis-Splicing genes of *R. arguta*; (b) Cis-splicing gene map of *R. parvifolia*; (c) Trans-splicing structure of the *rps*12 gene in *R. arguta*; and (d) Trans-splicing structure of the *rps*12 gene in *R. parvifolia*.

Supplementary Table S1. List of all 30 species, GenBank accession numbers and references for sequences used to construct phylogenetic trees (Figure 3).

| **Species** | **Accession number** | **References** |
| --- | --- | --- |
| *Hippophae rhamnoides* | NC035548 | Direct Submission |
| *Elaeagnus macrophylla* | KP211788 | Choi et al. 2015 |
| *Ceanothus divergens* | PQ858239 | Direct Submission |
| *Spyridium parvifolium* | ON534318 | Clowes et al. 2023 |
| *Spyridium phylloides* | NC064091 | Clowes et al. 2022 |
| *Spyridium spadiceum* | NC064095 | Clowes et al. 2022 |
| *Hovenia dulcis* | MT225403 | Li et al. 2020 |
| *Hovenia trichocarpa* | NC052875 | Li et al. 2020 |
| *Ziziphus jujuba* | MF781071 | Gao et al. 2017 |
| *Ziziphus mauritiana* | KY628304 | Huang et al. 2017 |
| *Ziziphus spina-christi* | KY628305 | Huang et al. 2017 |
| *Ventilago harmandiana* | NC065258 | Wanichthanarak et al. 2023 |
| *Ventilago leiocarpa* | OR750851 | Guo et al. 2024 |
| *Sageretia thea* | OR039202 | Zhan et al. 2024 |
| *Berchemia flavescens* | MK460212 | Zhu et al. 2019 |
| *Berchemia floribunda* | PQ463635 | Direct Submission |
| *Berchemia wilsonii* | KY926621 | Wang et al. 2018 |
| *Berchemia berchemiifolia* | NC037477 | Cheon et al. 2018 |
| *Rhamnella rubrinervis* | NC088754 | Direct Submission |
| *Rhamnella wilsonii* | NC066091 | Wang et al. 2024 |
| *Rhamnella martinii* | NC082535 | Direct Submission |
| *Frangula alnus* | NC068506 | Shi et al. 2023 |
| *Rhamnus leptacantha* | OR346919 | Huang et al. 2024 |
| *Rhamnus parvifolia* | PQ433220 | This study |
| *Rhamnus globosa* | ON009012 | Xie et al. 2020 |
| *Rhamnus erythroxylum* | PP706476 | Direct Submission |
| *Rhamnus cathartica* | OP374104 | Shi et al. 2023 |
| *Rhamnus arguta* | PQ433219 | This study |
| *Rhamnus taquetii* | MN901522 | Jin et al. 2020 |
| *Rhamnus heterophylla* | MT211599 | Li et al. 2020 |

**GenBank References**

Choi K S, Son O, Park S. 2015. The chloroplast genome of Elaeagnus macrophylla and trnH duplication event in Elaeagnaceae [J]. PLoS ONE, 10(9): e0138727. doi: 10.1371/journal.pone.0138727.

Clowes C, Fowler R, Fahey P, et al. 2023. Phylogeography and classification of Dusty Miller (Spyridium parvifolium; Rhamnaceae): a morphologically variable shrub from south-east Australia [J]. Plant Systematics and Evolution, 309 (15). doi: 10.1007/s00606-023-01851-5.

Clowes C, Fowler R M, Fahey P S, et al. 2022. Big trees of small baskets: phylogeny of the Australian genus Spyridium (Rhamnaceae: Pomaderreae), focusing on biogeographic patterns and species circumscriptions [J]. Australian Systematic Botany, 35: 95–119. doi: [10.1071/SB21034](https://doi.org/10.1071/SB21034).

Li M, Ye X, Bi H. 2020. Characterization of the complete chloroplast genome of two Hovenia species (Rhamnaceae) [J]. Mitochondrial DNA Part B: Resources, 5(2): 1731–1732. doi: 10.1080/23802359.2020.1749177.

Gao C M, Gao Y C, Liu X H. 2017. The complete genome of Ziziphus jujuba cv. Dongzao, an economic crop in the Yellow River Delta of China [J]. Mitochondrial DNA Part B: Resources, 2(2): 692–693. doi: 10.1080/23802359.2017.1383206.

Huang J, Chen R, Li X. 2017. Comparative analysis of the complete chloroplast genome of four known Ziziphus species [J]. Genes, 8(12): 340. doi: 10.3390/genes8120340.

Wanichthanarak K, Nookaew I, Pasookhush P, et al. 2023. Revisiting chloroplast genomic landscape and annotation towards comparative chloroplast genomes of Rhamnaceae [J]. BMC Plant Biology, 23(1): 59.

doi: 10.1186/s12870-023-04074-5.

Guo S, Li Z, Li C, et al. 2024. Assembly and characterization of the complete mitochondrial genome of Ventilago leiocarpa [J]. Plant Cell Reports, 43(3): 77. doi: 10.1007/s00299-023-03126-2.

Zhan M, Wang X, Chen W, et al. 2024. Complete chloroplast genome of Sageretia thea (Rhamnaceae), an ornamental fruit and medicinal tree [J]. Mitochondrial DNA Part B: Resources, 9(3): 376–380. doi: 10.1080/23802359.2024.2329667.

Zhu X F, Li Y, Lu Z. 2019. The complete chloroplast genome sequence of Berchemia flavescens (Rhamnaceae) [J]. Mitochondrial DNA Part B: Resources, 4(1): 1302–1303. doi: 10.1080/23802359.2019.1591240.

Wang Y H, Chen S Y, Zhang S D. 2018. Characterization of the complete chloroplast genome of Berchemiella wilsonii var. wilsonii (Rhamnaceae), an endangered species endemic to China [J]. Conservation Genetic Resources, 10: 39–41. doi: 10.1007/s12686-017-0760-y.

Cheon K S, Kim K A, Yoo K O. 2018. The complete chloroplast genome sequence of Berchemia berchemiifolia (Rhamnaceae) [J]. Mitochondrial DNA Part B: Resources, 3(1): 133–134. doi: 10.1080/23802359.2018.1431068.

Wang G, An J, Si J. 2024. The complete chloroplast genome sequence of Rhamnella wilsonii Schneid (Rhamnaceae) [J]. Mitochondrial DNA Part B: Resources, 9(4): 488–492. doi: 10.1080/23802359.2024.2339492.

Shi W, Hu S, Song W, et al. 2023. Uncovering the first complete chloroplast genomics, comparative analysis, and phylogenetic relationships of the medicinal plants Rhamnus cathartica and Frangula alnus (Rhamnaceae) [J]. Physiology and Molecular Biology of Plants, 29(6): 855–869. doi: 10.1007/s12298-023-01331-7.

Huang G, Li L, Wang X, et al. 2024. The complete plastid genome of Rhamnus leptacantha Schneid (Rhamnaceae) [J]. Mitochondrial DNA Part B: Resources, 9(1): 33–36. doi: 10.1080/23802359.2023.2300468.

Xie Y, Wang Z, Jiang X, Zhang X. 2020. The complete chloroplast genome of Rhamnus globosa (Rhamnaceae) [J]. Mitochondrial DNA Part B: Resources, 5(3): 2830–2831. doi: 10.1080/23802359.2020.1791010.

Jin D P, Park J W, Park J S, et al. 2020. The complete plastid genome of Rhamnus taquetii, an endemic shrub on the Jeju Island of Korea [J]. Mitochondrial DNA Part B: Resources, 5(1): 924–926. doi: 10.1080/23802359.2020.1719933.

Li B, Chen H, Chen J. 2020. The complete chloroplast genome of Rhamnus heterophylla (Rhamnaceae) [J]. Mitochondrial DNA Part B: Resources, 5(2): 1850–1851. doi: 10.1080/23802359.2020.1750987.
